# Performance of an Ultrasonic Ranging Sensor in Apple Tree Canopies

**DOI:** 10.3390/s110302459

**Published:** 2011-02-28

**Authors:** Alexandre Escolà, Santiago Planas, Joan Ramon Rosell, Jesús Pomar, Ferran Camp, Francesc Solanelles, Felip Gracia, Jordi Llorens, Emilio Gil

**Affiliations:** 1 Department of Agro-Forestry Engineering, University of Lleida, Av. Rovira Roure, 191. 25198 Lleida, Catalonia, Spain; E-Mails: SPlanas@eagrof.udl.cat (S.P.); JR.Rosell@eagrof.udl.cat (J.R.R.); Pomar@eagrof.udl.cat (J.P.); 2 Department of Agriculture, Livestock, Fisheries, Food and Environment, Center of Agricultural Mechanization, Generalitat de Catalunya, Av. Rovira Roure, 191. 25198 Lleida, Catalonia, Spain; E-Mails: Ferran.Camp@gencat.cat (F.C.); FSolanelles@gencat.cat (F.S.); FelipJ.Gracia@gencat.cat (F.G.); 3 Department of Agro-Food and Biotechnology Engineering, Polithecnical University of Catalonia, Av. Canal olímpic, 15. 08860 Castelldefels, Catalonia, Spain; E-Mails: Jordi.Llorens.calveras@upc.edu (J.L.); Emilio.Gil@upc.edu (E.G.)

**Keywords:** ultrasonic sensor, distance measurements, apple tree orchard, ultrasonic interferences

## Abstract

Electronic canopy characterization is an important issue in tree crop management. Ultrasonic and optical sensors are the most used for this purpose. The objective of this work was to assess the performance of an ultrasonic sensor under laboratory and field conditions in order to provide reliable estimations of distance measurements to apple tree canopies. To this purpose, a methodology has been designed to analyze sensor performance in relation to foliage ranging and to interferences with adjacent sensors when working simultaneously. Results show that the average error in distance measurement using the ultrasonic sensor in laboratory conditions is ±0.53 cm. However, the increase of variability in field conditions reduces the accuracy of this kind of sensors when estimating distances to canopies. The average error in such situations is ±5.11 cm. When analyzing interferences of adjacent sensors 30 cm apart, the average error is ±17.46 cm. When sensors are separated 60 cm, the average error is ±9.29 cm. The ultrasonic sensor tested has been proven to be suitable to estimate distances to the canopy in field conditions when sensors are 60 cm apart or more and could, therefore, be used in a system to estimate structural canopy parameters in precision horticulture.

## 1. Introduction

Ultrasonic sensors have been used for many purposes in agriculture for more than 40 years. One of these applications has been the detection and ranging to extract geometric information from fruit tree canopies. The first developments in this area were related to the application of plant protection products such as pesticides and fungicides in fruit orchards. Once doses started to be adjusted according to the amount of vegetation to be treated [1,2], some researchers began to develop electronic systems to quantify the geometric parameters of canopies. The first proposals to estimate canopy volume used several ultrasonic sensors mounted in a vertical mast [3] or mounted on a sprayer [4] driven by a tractor. However, the state-of-the-art of the application technologies did not allow this information to be used in real time. Instead, some sprayer patents [5,6] and scientific works [7–10] were published mounting ultrasonic sensors to only detect the presence of canopy in order to exclusively spray when vegetation was in front of the nozzles. Another application of ultrasonic sensors is the one designed to spray citrus canopies at a constant given distance [11]. The nozzles are mounted on a moving arm controlled to follow the contour of the canopy according to the information provided by a sensor. In this work an accuracy analysis can be found when sensors are placed 50 cm and 75 cm apart. The average error in their citrus grove distance measurements is 11.40 cm. However, information is not provided about the importance of the effect of the foliage on the ultrasonic cone or the effect of interferences in such an error. The same authors developed a sprayer able to spray three different flow rates according to a canopy width estimation made with an ultrasonic sensor [12]. There was no spraying when there was no vegetation; half the flow rate was sprayed when little vegetation was detected and the full flow rate was sprayed when canopy width was higher than a predefined threshold. This design led the way to a continuous variation of the flow rate according to the variability of the vegetation along fruit orchard, vineyard or citrus grove rows [13–18]. A lot of research has been carried out to automatically measure canopy dimensions in citrus. First works were focused on comparing manual volume estimations with measurements performed with ultrasonic and lidar sensors [19]. Results showed that the estimations of ultrasonic and lidar sensors correlated fairly well, whereas the correlation with manual estimations was lower. The authors attributed the differences with manual measurements to the higher sampling resolution achieved with the sensors. The ultrasonic system consisted of a mast with 10 ultrasonic sensors per side. In order to avoid signal interferences, alternated sensors were fired in different groups sequentially. This system was later used to map canopy volumes in citrus orchards by fitting a DGPS receiver [20–22]. The authors observed that in less dense trees bigger differences were found between manual and sensor estimations. These authors used canopy volume information to adjust the fertilizer dose rate [23,24] and to estimate the fruit yield in citrus groves [25]. In relation to the vertical sampling resolution, lidar sensors provide much more information than an array of ultrasonic sensors resulting in a more accurate estimation of canopy parameters [26–29]. Another application has been the use of ultrasonically estimated canopy volumes, together with other information, to define management zones in citrus groves [30,31]. Sources of errors in estimating canopy volumes in citrus with the previously described system in comparison with manual measurements have also been studied [32]. The most important source of error in their system was the inaccuracy of the DGPS receiver in estimating ground speed followed by the effect of air temperature on the time of flight of sound waves and, therefore, on the sensors distance estimation. A last source of error was the deviations in the driving path. Nonetheless, no information was provided about the effect of canopy structure on the sensor output or other parameters potentially affecting the reflectance of the ultrasonic waves. Recently, an ultrasonic sensor has been studied in order to determine its accuracy when measuring distances to simulated canopies of field crops [33]. The results are promising for field crops but difficult to extrapolate to tree crops due to differences in canopy structure between crops.

Most of the referenced applications of ultrasonic sensors in canopy characterization are focused on correlating manual estimations of width or volume with the results obtained by using the sensors. These works did not provide information about the interaction between the sound wave and the canopy itself and how can it interfere in the estimations of ultrasonic sensors. In ranging applications, ultrasonic sensors are intended to estimate distances to objects with solid surfaces in order to get specular reflections of the acoustic waves. The surface of an apple tree canopy is made up of small leaf surfaces placed in different orientations. Consequently, the reflection of ultrasonic waves is more diffuse than specular and this can strongly affect estimated distances.

In this paper a commercial ultrasonic sensor model is analyzed to validate its suitability, performance and reliability in an apple orchard and for a better understanding of the sensing process. Trials have been performed in laboratory and field conditions in a stationary way in order to assess its ability to estimate both distances to the canopy and the effect of possible interferences coming from other adjacent sensors. This study is the first step in a larger work that would use this type of sensors to estimate canopy parameters such as cross section areas or canopy volume in fruit tree crops in the framework of precision horticulture.

## 2. Materials and Methods

One ultrasonic sensor and a data acquisition system were used in the laboratory and field trials. The sensors were mounted on a vertical aluminum mast on a mobile platform, which was also used to carry the acquisition system and the batteries to supply the required voltage to run the electronic devices.

### 2.1. Sensor and Data Acquisition

**Figure 1.**
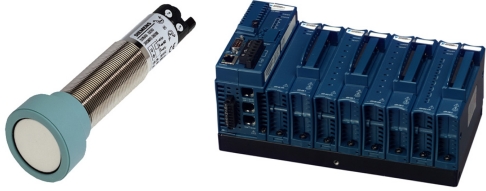
Ultrasonic sensor used in this work (**left**) model Sonar Bero PXS400 M30 K3 (Siemens AG, Munich, Germany). Electronic device for data acquisition and control of the trials (**right**) model compactFieldPoint 2120 (National Instruments, Austin, TX, USA).

The ultrasonic sensor selected to carry out the trials is a Sonar Bero PXS400 M30 K3 (Siemens AG, Munich, Germany, Figure 1 left). A different model of the same series has already been used in field works in citrus with an increased detection limit [11]. The sensor is considered to be robust enough in terms of working temperature (the sensor has an internal temperature sensor to compensate the effect of this parameter in the distance estimation), degree of protection, shock and vibration resistance. Since row spacing in fruit tree orchards is usually 5 m or less and the sensor is thought to be placed in the center of the alleys, the chosen sensing range was from 40 cm to 300 cm. However, any sensing range modification results in differences in the emitted ultrasonic cone angle: the shorter the range, the narrower the ultrasonic cone angle. Due to this difference with the sensor used in citrus and the differences in the crop itself, it was considered important to assess the performance of this sensor in an apple orchard before being used in canopy characterization. Other specifications of the sensor can be found in Table 1.

The acquisition system (Figure 1 right) consists of a PAC (Programmable Automation Controller) model compactFieldPoint (National Instruments, Austin, TX, USA). This device mounts an analog input module where the sensors are connected. This device can be operated from a laptop running specifically designed pieces of software programmed using LabVIEW (National Instruments).

**Figure 2.**
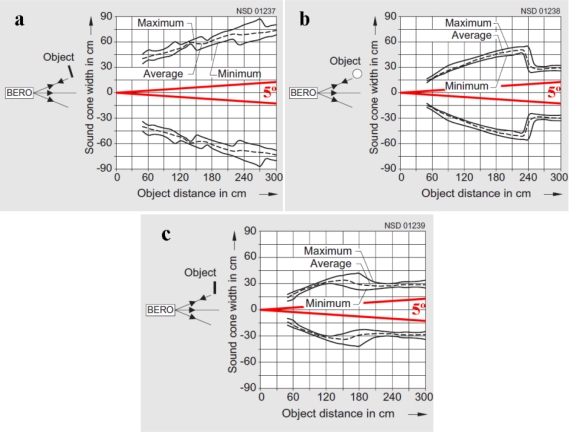
Sound cone diagrams for different types and orientations of targets with a superimposed 5° beam angle cone projection in red. *Source*: Siemens AG. Simatic sensors catalog: sensors for factory automation, FS 10, 2008.

An important parameter to be taken into account is the beam angle. In the specification sheet delivered together with the sensor, the information provided related to this parameter is as follows: *in the operating range, objects are sensed reliably within a sound cone angle of 5°. Under good reflection conditions, objects can also be sensed outside*. In a more detailed catalog, the manufacturer provides information about the shape and size of the sound cones depending on the object to be detected (Figure 2). The targets are a 10 × 10 cm squared plane object and an 8 cm diameter cylindrical object. To obtain these diagrams, the squared target is placed both aligned, with the most optimum reflection (Figure 2(a)), and perpendicularly to the ultrasonic cone axis (Figure 2(c)). The cylindrical target cone diagram is described in Figure 2(b). An ultrasonic cone projection with a 5° beam angle has been superimposed to the diagrams. It is clear that the target itself and its relative position to the sensor can greatly affect the detection footprint of the sensor.

Ultrasonic sensors are also very sensitive to interfering sonic waves coming from nearby sensors. The manufacturer offers two methods to synchronize several sensors in order to avoid inaccurate readings due to interferences of sonic echoes sent from another sensor. However, both methods present big disadvantages. In one method the object cannot be assigned to a particular sensor. In the other, longer response times are required because each sensor is only active briefly and then has to wait until all the other sensors in the circuit have emitted. The latter, causes the array of sensors not to obtain simultaneous readings, what could cause inaccurate estimations of cross-sectional areas of tree rows. These solutions could be useful in a stationary system but are not satisfying at all when the sensors are boarded in a moving platform. In this last situation, a minimum distance between active sensors must be found in order to obtain the highest vertical resolution to better estimate canopy parameters with the least effect of interferences.

### 2.2. Laboratory Distance Measurement Trial

**Figure 3.**
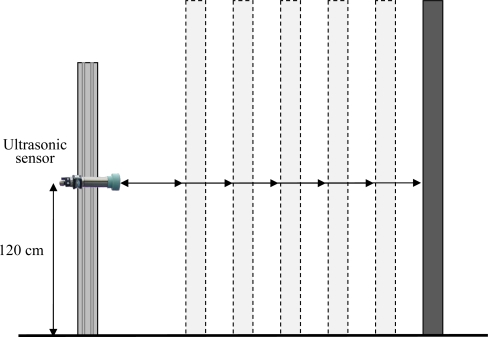
Experiment layout for the laboratory distance measurement trial.

The laboratory distance measurement trial was carried out at the *Centre de Mecanització Agrària* (Center of Agricultural Mechanization) of the Department of Agriculture, Livestock, Fisheries, Food and Environment of the *Generalitat de Catalunya* (Catalan Government) in Lleida, Catalonia, Spain. The trial was designed to assess the capabilities of the ultrasonic sensor under ideal conditions using a hard, flat, 10 cm × 100 cm metallic target (bigger than the minimum dimensions recommended by the manufacturer). The mobile platform carrying the sensor was stationed and the target was placed at different known distances to the sensor (Figure 3). 2,000 readings were stored at each specific distance ranging from 45 cm to 285 cm in steps of 15 cm. All measurements were performed in stationary and no wind conditions.

The statistical analysis consisted of fitting a linear regression model (Equation (1)) in order to establish the existence of a functional relationship between the distance to the sensor and the sensor analog output. If possible, a regression line would be defined according to the least squares method. The quality of the fitting was assessed by analyzing the measured distance *vs*. the sensor output and the measured distance *vs*. the residual scatter diagrams and by analyzing the coefficients of correlation (r) and determination (R^2^), the root mean square error (RMSE) and the significance of the model.

(1) $$\hat{d}={\hat{\beta }}_{0}+{\hat{\beta }}_{1}\;v$$

where d̂ is the estimated distance; β̂_0_ is the estimation of the intercept; β̂_1_ is the estimated parameter multiplying the regressor; v is the independent variable, that is, the sensor output.

### 2.3. Field Trials

**Figure 4.**
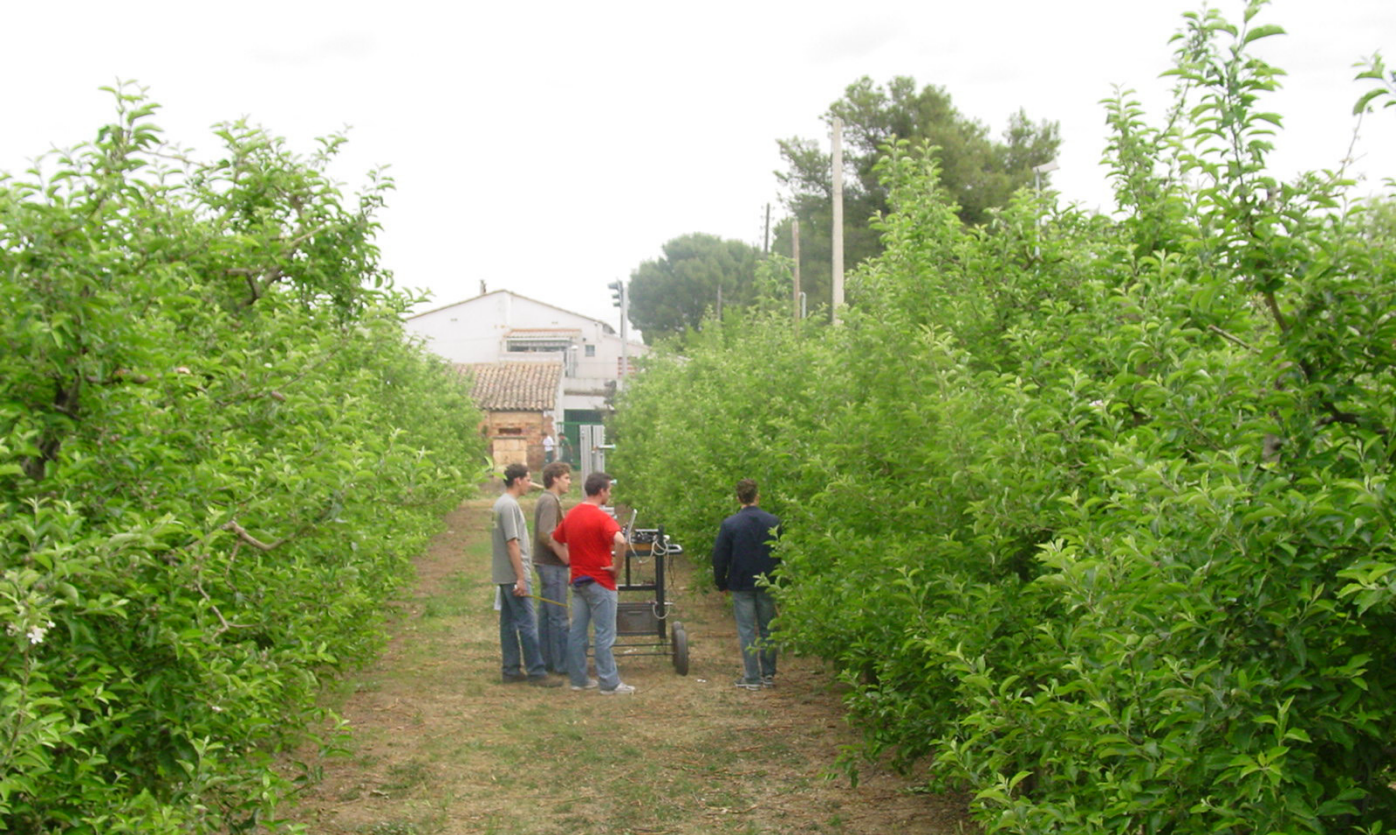
*Malus domestica*, Borkhausen cv. ‘Golden Delicious’ apple orchard where the field trials were carried out.

Both distance measurement and interference trials were conducted in an *Malus domestica*, Borkhausen cv. ‘Golden Delicious’ apple orchard (Figure 4) at the campus of the *Escola Tècnica Superior d’Enginyeria Agrària* (School of Agricultural and Forestry Engineering) of the *Universitat de Lleida* (University of Lleida) in Lleida, Catalonia, Spain. The characteristics of the orchard are listed in Table 2. All measurements in the field trials were performed under stationary and no wind conditions in order to only assess the capability of the sensor without introducing other error sources.

#### 2.3.1. Distance Measurement Trial

**Figure 5.**
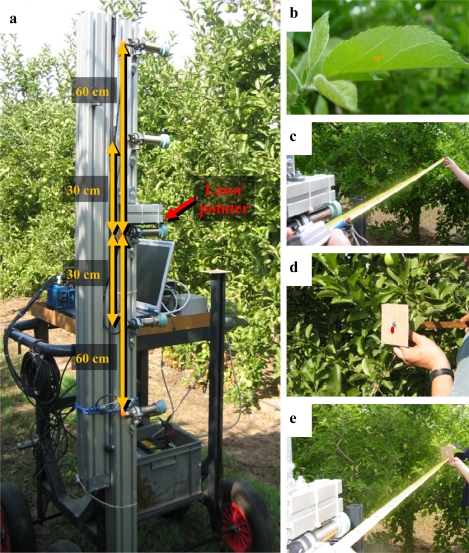
Test platform designed for both the field distance measurement and the interference trials (**a**) and sequence of the procedure for distance assessment (**b**, **c**, **d** and **e**).

**Figure 6.**
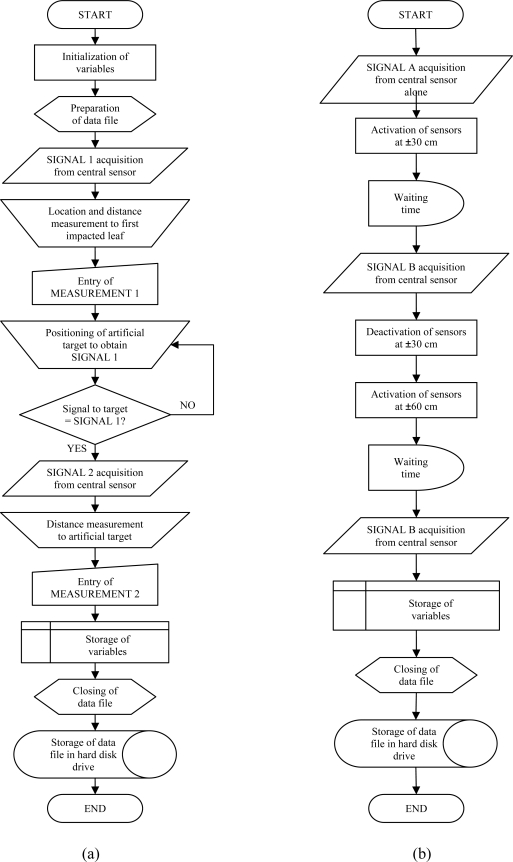
Flux diagrams of the programs designed for the field distance measurement (**a**) and the interference (**b**) field trials.

The aim of this trial was to compare the analog output of the sensor when measuring different distances to the canopy with the output when measuring the distance to an ideal target. To this purpose, a methodology and a test bench were designed and implemented (Figure 5). The first step of the methodology was to fix the test bench and record the output of the central sensor (Figure 5(a)). Afterwards, the first detected leaf is located with the help of a laser pointer parallel to the ultrasonic sensor (Figure 5(b)). The next step was to manually measure the distance from the sensor to that specific leaf with a tape (Figure 5(c)). After that, an artificial 10 cm × 10 cm cardboard target is placed at a distance from the sensor so the same previously recorded sensor output for the canopy is obtained (Figure 5(d)). Finally, a tape is used to manually measure the distance to the cardboard (Figure 5(e)). The distance to the artificial target is used to validate the data obtained when measuring the distance to the leaves. In Figure 6(a), a flux diagram of the process is shown. This process was repeated 168 times in different zones of the orchard in order to give statistical significance to the trial.

The statistical analysis of the field data is analogous to the one carried out for the laboratory trial in terms of the type of fitted model (Equation (1)) and its quality assessment. The fitted linear model in field conditions was compared to the one obtained in laboratory conditions in order to assess its similarities and differences.

#### 2.3.2. Interference Trial

The aim of the trial was to assess the effect of adjacent sensors working simultaneously with the central one. The manufacturer suggests that the user experimentally determine the distance between sensors to avoid interferences in situations other than specular sonic reflection. The interference trial has been carried out with the same test platform used in the distance measurement trial. In this case, the platform was fitted up with two more pairs of ultrasonic sensors placed at ±30 cm and ±60 cm around the central ultrasonic sensor in the vertical plane (Figure 5).

These distances have been chosen considering possible future applications of the sensor related to canopy characterization. In order to better estimate parameters such as cross sectional canopy areas or canopy volume, the closer the sensors could be the higher the vertical sampling resolution. In the previously referred analysis carried out in the citrus grove [11], separations between sensors were 50 cm and 75 cm. In this research work, the effect of interferences in a much closer set up than 50 cm was assessed (±30 cm) as well as an intermediate value between 50 cm and 75 cm (±60 cm) in order to determine the minimum reliable sensor separation.

To this purpose, the methodology described in the flux diagram of Figure 6(b) was designed and implemented in a piece of software so that the output of the central sensor alone could be compared with the output obtained when working together with the sensors located at ±30 cm and with the ones located at ±60 cm. Activation and deactivation of adjacent sensors was achieved by switching on and off the sensors’ power supply accordingly. The process was repeated 113 times in different zones of the orchard in order to give statistical significance to the trial. The effect of interferences was assessed by analyzing the scatter diagrams of the central sensor output when working alone or simultaneously with adjacent sensors and by analyzing the histogram and the cumulative frequencies.

## 3. Results and Discussion

### 3.1. Laboratory Distance Measurement Trial

The total number of observations was 34,000. However, fifteen of them were removed as they were considered outliers. In Table 3 it is clearly seen that the correlation between the output signal of the sensor and the target distance is very strong. The fitted model can explain a 99.9% of the variability of the response. The RMSE is very low and gives an idea of the good fitting of the model. The significance of the fitted model, represented by the p value of the analysis of variance of the model, is very high. The p value leads to refuse the null hypothesis which assumes that all parameters are equal to zero. Therefore it is possible to find a significant parameter for the regression line (Equation (2)).

(2) d=297.07−26.36 v

where d is the estimated distance, expressed in cm and v is the output voltage signal of the sensor, expressed in V.

**Figure 7.**
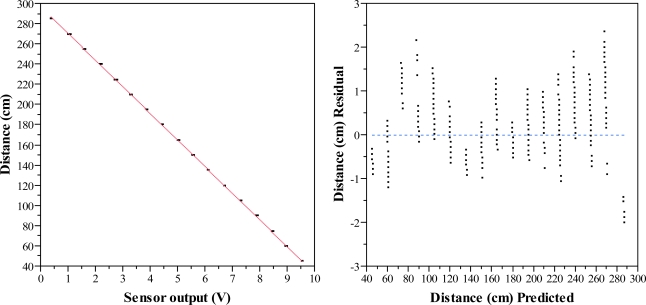
Scatter diagram of sensor output and distances (**left**) and residuals of predicted distances (**right**) for the laboratory distance measurement trial between 45 cm and 285 cm in steps of 15 cm. The red line in the diagram is the fitted regression line.

The regression line, as well as results of the distance measurement laboratory trial, is shown in Figure 7. The residual scatter plot demonstrates that there is not a clear structure for the residuals so we can accept the fitted model. The average error in absolute value is 0.53 cm.

### 3.2. Field Trials

#### 3.2.1. Distance Measurement Trial

**Figure 8.**
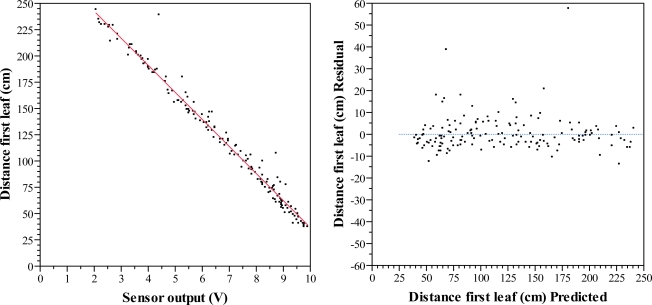
Scatter diagram of sensor output and distances to the first leaf (**left**) and residuals of the predicted distances to the first leaf (**right**) for the field distance measurement trial.

Total number of observations was 168. Ten observations were removed as they were considered abnormal when compared with their equivalent measurement to the 10 cm^2^ cardboard target. In Table 4 it is seen that the correlation between both variables is still very strong. The fitted model can explain 98% of the variability of the distance to the first leaf. The main difference with the model fitted under laboratory conditions is the RMSE. While an artificial plane target produces a good echo, the increase of RMSE in field conditions tells that variability in the response is higher. Even though, the fitted model is still highly significant, as stated by the p value. So it is possible to find a significant parameter for the regression line (Equation (3)). When comparing Equations (2) and (3), the difference between the two parameters is rather small while intercepts differ 2.79 cm. The regression line, as well as results of the distance measurement laboratory trial, is shown in Figure 8. The residual scatter plot demonstrates that there is not a clear structure for the residuals so we can accept the fitted model.

(3) d=294.28−25.82 v

where d is the estimated distance, expressed in cm and v is the output voltage signal of the sensor, expressed in V.

**Figure 9.**
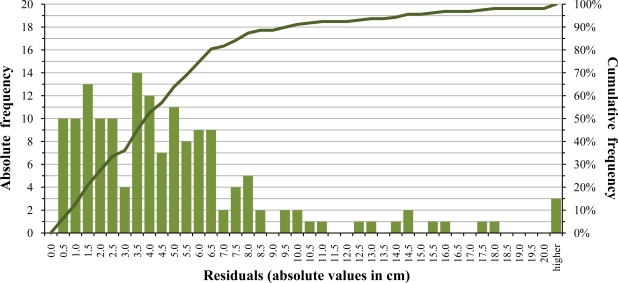
Histogram and cumulative frequency of residuals in absolute value for the field distance measurement trial.

The average absolute error is 5.11 cm. A frequency analysis of the residuals in absolute value shows that the 20.9% of observations have an absolute error smaller or equal to 1.5 cm, when compared with the regression line established by the fitted linear model. A 52.5% of observations have an absolute error smaller or equal to 4 cm, a 74.7% smaller or equal to 6 cm and a 91.1% smaller or equal to 10 cm (Figure 9). This decrease of accuracy in field conditions has an effect on the final estimated canopy parameter. For example, when estimating canopy cross sectional areas with one ultrasonic sensor, a few centimeters may have a relatively higher effect than using two or more sensors at different heights.

In order to compare the model for laboratory conditions with the one obtained in field conditions, a numerical simulation has been carried out. In Table 5 the estimated distances for a sensor output when using the field model, the laboratory model and its difference are shown. The biggest differences are found at both ends of the sensing range. At the central part of the range differences are very small. The RMSE of these differences is 1.69 cm, which is indeed very small.

**Figure 10.**
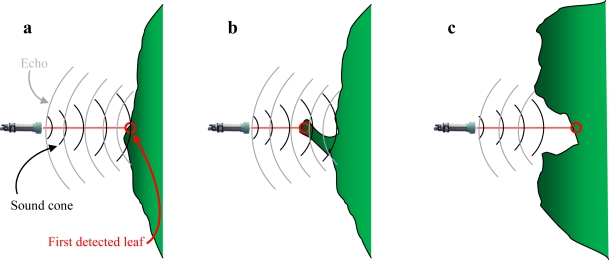
Interaction possibilities between ultrasonic waves and canopy.

However, canopy surface characteristics provoke a higher variability in distance estimations. This is caused by the capacity of leaves to generate a sufficient echo for the sensor to acknowledge it. In Figure 10 some diagrams to better understand how canopy can interact with sonic waves are shown.

Most times a single leaf is not enough to generate a sufficient echo due to its reduced dimensions and/or to its orientation. To estimate a correct distance, the sensor needs to detect a group of leaves approximately placed in the same vertical plane (Figure 10(a)).

If this group of leaves is not enough to produce a suitable echo, it will not be produced there, but rather some distance further away where a bigger mass of leaves can generate one (Figure 10(b)). In such a situation, the measured distance between the sensor and the canopy would be shorter than the one estimated by the model and thus the measurement would be below the regression line (Figure 8 left).

Alternatively, it is possible that canopies present gaps in the outer layer of leaves. When a sensor is aligned with one of these gaps it could happen that the echo is produced by the surrounding leaves of the gap instead of by the leaf mass inside the gap (Figure 10(c)). In such a situation, the measured distance between the sensor and the canopy would be greater than the one estimated by the model and thus the measurement would be over the regression line (Figure 8 left). This situation has to do with the shape of the sound cone and with the orientation and dimensions of leaves. The manufacturer provides three different diagrams according to the shape and orientation of the target (Figure 2). In all these three diagrams the sensing area is sensibly bigger than the obtained with a 5° sound cone angle, the aperture considered to reliably detect an object. Therefore, the sensing footprint of ultrasonic sensors varies according to the distance to the object. The further the canopy, the lower the capacity to detect gaps in that canopy is. However, it is not easy to determine the sound cone under field conditions when sensing leaves because of the multiple parameters to be taken into account. Thus, the footprint drawn in Figure 2 for a 5° sound cone is only an approximation of what the minimum gap dimensions are to be detected with this kind of sensors.

#### 3.2.2. Interference Trial

**Figure 11.**
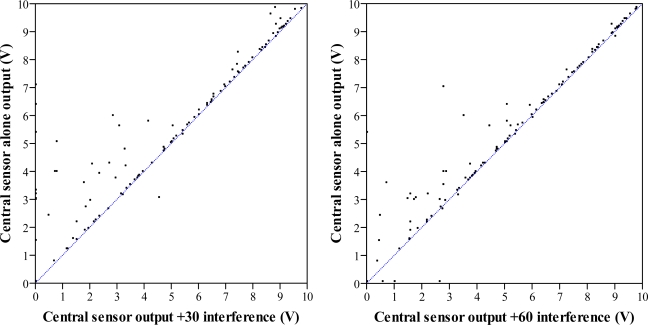
Scatter diagram of central sensor alone output and simultaneously working with adjacent sensors at ±30 cm (**left**) and with adjacent sensors at ±60 cm (**right**) during the field interference trial. Blue lines represent bisectors.

A total of 113 observations were carried out according to the methodology described in the flux diagram in Figure 6(b). Adjacent sensors placed at ±30 cm and ±60 cm do interfere the output signal of the central sensor, as seen in Figure 11. When using sensors separated ±60 cm, the first 100 cm are less sensitive to interferences than the rest of the sensing range, while the interferences caused by sensors at ±30 cm affect all the sensing range. In general, the further the canopy, the bigger the effects provoked by nearby sensors are. The consequence of interferences is that the output signal of interfered sensors may sporadically be higher than it should be. According to the field distance estimation (Equation (3)) interferences would tend to decrease the predicted distance to the canopy which implies an overestimation of its width. In the case of using ultrasonic sensors to determine the canopy volume to adjust the dose rate of plant protection products, overestimation of canopy volumes with interferences is not as negative as underestimation. Underestimations could decrease the efficacy of the treatment and create resistance of pest to certain active ingredients.

**Figure 12.**
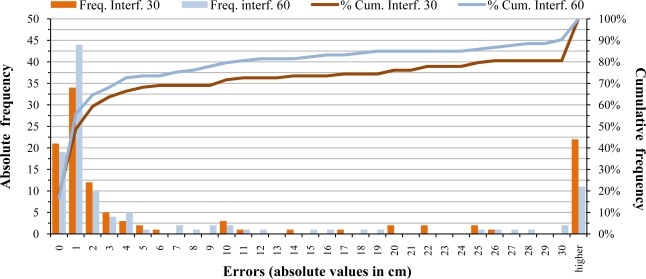
Histogram and cumulative frequencies of errors caused by interferences from adjacent sensors at ±30 cm and at ±60 cm for the field interference trial.

In Figure 12, errors caused by interferences from adjacent sensors at ±30 cm and at ±60 cm are shown. In both cases, most measurements are practically unaffected by interferences, but some of them are greatly affected. The average error when working with sensors separated by 30 cm is ±17.46 cm. In case of a separation of 60 cm, the average error is ±9.49 cm.

According to the results and to the information provided by the manufacturer, the more apart the sensors are, the lower the effect of interferences is. However, a balance should be found between the sensor separation and the highest vertical resolution (maximum number of sensors to estimate the canopy vertical outline in a more accurate manner). Anyhow, it would be possible to overcome the effects of interferences by using an appropriate filter, *i.e.*, a median filter [11], to avoid drastic changes in sensor output in short time intervals.

## 4. Conclusions

The tested ultrasonic sensor is able to accurately estimate distances under laboratory conditions with an average error of ±0.53 cm. When used under field conditions, the distance estimation equation should be adapted to better estimate distances to the canopy. However, differences with the laboratory estimation equation are relatively small, considering other possible sources of error.

The variability in distance estimations in field conditions in an apple orchard clearly increases in relation to what was obtained in laboratory with artificial targets. As a consequence of this, the average error is ±5.11 cm. The effect of interferences is higher when sensors are 30 cm apart with an average error of ±17.46 cm. When sensors are separated 60 cm, the average error is ±9.29 cm. Sensors should thus be separated more than 60 cm in order to avoid high interference effects.

Ultrasonic sensors like the one tested and reported in this paper have been proven to be suitable to estimate distances to the canopy in field conditions. Results could be extrapolated to other apple crop varieties and other species such as pear crops where canopy structures and leaf dimensions are similar.

However, it has to be taken into account that the increase of variability due to the characteristics of the canopy surface and the ultrasonic working principle reduces the accuracy of the estimations and that the effect of interferences can be important when adjacent sensors are too close.

## Figures and Tables

**Table 1.** Specifications of the ultrasonic sensor used in this work (model Sonar Bero PXS400 M30 K3, Siemens AG, Munich, Germany).

**Parameter**	**Value**
Sensing range	40 cm to 300 cm
Target dimensions for max. meas. dist.	5 cm × 5 cm
Response time	50 ms to 200 ms
Accuracy	±1.5%
Resolution	1 mm
Beam angle	Approx. 5°
Sensor output	0 VDC a 10 VDC
Ultrasound frequency	120 kHz
Weight	approx. 150 g
Ambient temperature (compensation)	−25 °C to +70 °C
Operating voltage	20 VDC to 30 VDC
Vibrating stress	11 to 55 Hz, 1 mm amplitude
Shock stress	30 g, 18 ms
Degree of protection	IP 65

**Table 2.** Characteristics of the *Malus domestica*, Borkhausen cv. ‘Golden Delicious’ apple orchard where the field trials were carried out.

**Parameter**	**Value**
Phenological stage	BBCH 76
Row spacing	5.00 m
Tree spacing	1.60 m
Representative canopy width	1.75 m
Representative tree height	3.75 m

**Table 3.** Summary of the fitted linear model to estimate distances to an artificial target from the sensor output in laboratory conditions.

**Parameter**	**Value**
Observations	33,985
Coef. of determination (*R^2^)*	0.999
Coef. of correlation *(r)*	−0.999
RMSE (cm)	0.699
p value of the model	<0.0000

**Table 4.** Summary of the fitted linear model to estimate distances to the first leaf from the sensor output in field conditions.

**Parameter**	**Value**
Observations	158
Coef. of determination (*R^2^)*	0.980
Coef. of correlation *(r)*	−0.990
RMSE (cm)	8.107
p value of the model	<0.0001

**Table 5.** Numerical simulation comparing distance estimations according to field and laboratory measurements.

**Output of the sensor (V)**	**Field distance estimation (cm)**	**Laboratory distance estimation (cm)**	**Difference (cm)**
0.00	294.28	297.07	−2.79
1.00	268.46	270.71	−2.26
2.00	242.63	244.36	−1.72
3.00	216.81	218.00	−1.19
4.00	190.98	191.64	−0.66
5.00	165.16	165.29	−0.13
6.00	139.34	138.93	0.40
7.00	113.51	112.58	0.94
8.00	87.69	86.22	1.47
9.00	61.86	59.86	2.00
10.00	36.04	33.51	2.53
